# Highly efficient, chemically defined and fully scalable biphasic production of vaccine viruses

**DOI:** 10.1186/1753-6561-5-S8-O1

**Published:** 2011-11-22

**Authors:** Ingo Jordan, Volker Sandig

**Affiliations:** 1 ProBioGen AG, 13086 Berlin, Germany

## 

Vectorial vaccines are predicted to yield novel therapeutic and protective approaches. They consist of recombinant live carriers that express antigen from an unrelated pathogen in the recipient. Promising viral carriers are host-restricted pox viruses that trigger a strong immune response without ability to replicate in the human organism. A block in replication is an important safety feature that allows application even in immunocompromized recipients. However, with this type of attenuation there is not even limited amplification of the vector at the site of infection and therefore very high numbers of infectious units have to be given per dose for full efficacy. Hence, if such vectors are to be used in global vaccine programs highly efficient production processes will be required. Furthermore, to combat diseases such as AIDS, hepatitis C, tuberculosis, or malaria with these vectors, millions of the concentrated vaccine units will have to be provided annually. Any production process therefore should also be scalable and preferrably transferrable to newly industrialized countries. Latter requirement demands a robust process independent of the complex logistics and uncertainties associated with primary chicken cells, the current industrial substrate for these pox viruses and certain other vectors.

We believe that we have solved most of the upstream challenges with a chemically defined suspension culture production process for three disparate members of the highly attenuated poxviruses [1]: modified vaccinia Ankara (MVA), fowlpoxvirus (FPV) and canarypoxvirus ALVAC. The process is independent of primary material and based on the continuous duck suspension cell line AGE1.CR specifically created as a vaccine substrate [2].

In contrast to production of influenza virus that readily replicates in the cell proliferation medium [3], development of a production process for poxviruses was surprisingly complicated and involves media formulations matched to two distinct phases, cell proliferation and virus production. Our process was adjusted to the different kinetics and requirements of the three examined viruses, and was studied in Wave and disposable bioreactors up to 50 L scale. For MVA, titers in the crude lysate without any processing reliably exceed the critical threshold of 10^8^ pfu/mL and often are in the range of 5 × 10^8^ to 2 × 10^9^ pfu/mL.

One hallmark of the biphasic process described here is controlled formation of suspension cell aggregates at the transition from cell proliferation to virus production. Such aggregate formation was achieved with distinct chemically defined media harmonized such that a highly efficient, robust and industrial biphasic processes was obtained that does not require perfusion, medium replacement or microcarriers.

**Figure 1:**
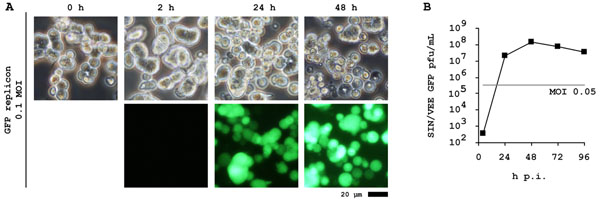
Biphasic process adapted to alphavirus replicons.

In addition to poxviruses, this approach was successful in production of an unrelated RNA vector and also for this reason we believe that we have developed a more general principle for scalable production of viruses that may benefit from cell-to-cell contacts: In the background of the AGE1.CR cell lines, packaging cells were created for SIN/VEE-chimeric alphavirus replicons [4]. The packaging cells were transfected for stable trans-complementation of envelope and capsid proteins from separate expression cassettes. Production of replicons was efficient using adherent (serum-dependent) cultures but only moderately efficient with suspension cultures of the packaging cells. After transfer and optimization of the pox virus production process to replicon-induced packaging cell cultures (shown in figure 1) we obtained yields beyond 10^8^ pfu/mL.

*Appearance of suspension packaging cells just prior to infection and at various time points during virus production is shown in* (*A*)*. Note induction of aggregates in presence of virus production medium and cytopathic effect 24 h post induction. Yields of replicon is shown in* (*B*) *with peak titers 24 h to 48 h post induction.*

In summary, superior yields were obtained for unrelated viral vectors using separate chemically defined media for proliferation and virus production. The media are matched to allow highly efficient, robust and industrial biphasic processes that do not require perfusion or medium replacement.
